# Fracture threshold of tooth roots and stress analysis of surrounding tissue during the extraction of impacted mandibular third molars using dental elevators

**DOI:** 10.1186/s12903-025-06744-2

**Published:** 2025-10-08

**Authors:** Zhengfen Li, Yijun Liu, Jiangling Sun, Meiyan Rong, Cheng Niu, Xubo Duan, Wei Yang, Mingkun Liu

**Affiliations:** 1 Guizhou University Medical College, Guiyang, Guizhou 550025 China; 2 School of Civil Engineering, Guizhou University, Guiyang, Guizhou 550025 China; 3 Guiyang Hospital of Stomatology, Guiyang, Guizhou 550002 China; 4 School of Stomatology, Zunyi Medical University, Zunyi, Guizhou 563000 China; 5 Qilu Medical College, Zibo, Shandong 255000 China

**Keywords:** Impacted mandibular third molar, Dental elevator, Fracture threshold, Biomechanical modeling, Surgical extraction

## Abstract

**Background:**

This biomechanical study aimed to investigate root fracture thresholds and stress distributions in surrounding tissue while extracting three types of impacted mandibular third molars (horizontal, vertical, and mesioangular impactions) under varying dental elevator loading conditions.

**Methods:**

Mechanical tensile testing was conducted to determine the anisotropic properties and maximum fracture load of extracted teeth. Three-dimensional finite element models, reconstructed from cone beam computed tomography (CBCT) data, were used to simulate elevator-assisted extraction processes. Stress distributions and root fracture thresholds were analyzed under three loading modes (wedge, lever, and rotational force) at different abduction angles.

**Results:**

Horizontal impactions demonstrated the lowest fracture resistance under lever forces (38.7–50.3 N), while vertical impactions exhibited the highest thresholds (110.3–342.2 N). Wedge forces showed angular dependence inversely correlated with fracture thresholds for horizontal impactions (76–174 N). Rotational moments maintained relatively stable thresholds across all impaction types (X-axis: 21–32 N·m, Y-axis: 20–40.8 N·m, Z-axis: 16.3–33.5 N·m). Surrounding tissue stress decreased with increasing abduction angles under lever and wedge forces (*p* < 0.05) but no significant directional correlation was observed under rotational moments.

**Conclusions:**

Dentin anisotropy and elevator angulation significantly influence fracture mechanics. Clinical protocols should prioritize lever forces for vertical impactions (safety margin > 100 N), with wedge and rotational force more effective for horizontal impaction. The findings provide an important theoretical basis for oral surgeons in extracting impacted third molars and for the subsequent development of surgical path planning and intelligent reasoning systems by the research group.

**Supplementary Information:**

The online version contains supplementary material available at 10.1186/s12903-025-06744-2.

## Background

The third molars frequently fail to erupt normally due to the obstruction of surrounding tissues, leading to impacted third molars. In clinical practice, preventive extraction is commonly adopted [1]. During the extraction of impacted third molars, the dental elevator plays a crucial role as a key instrument. The angle and magnitude of the applied force directly influence the success of the procedure. Improper force application can result in various complications, such as tooth root fracture and damage to surrounding tissues [2, 3]. Therefore, precise control of the applied load is essential for ensuring surgical predictability and preventing complications. However, due to the complex position and shape of impacted third molars and their surrounding tissues, the technical requirements for extraction are high, making it far from a simple direct extraction [4]. This process often relies on the clinical experience of professional doctors, which introduces subjectivity and uncertainty. For young or inexperienced doctors, accurately recognizing and predicting the risk of root fracture during the extraction of impacted third molars poses a significant challenge [5]. Consequently, biomechanical analysis is of vital importance.

Biomechanical research is grounded in Newtonian mechanics and continuum mechanics, employing both theoretical and experimental approaches. Theoretical research primarily utilizes finite element analysis (FEA), a discretized numerical calculation technique. FEA divides complex geometric structures into a finite number of elements and obtains the global behavior of the entire structure by solving the responses of these local elements [6, 7]. This method offers repeatable simulations and avoids ethical restrictions [8]. Since Thresher et al. first applied FEM to study oral problems in 1973 [9], this approach has become a major tool for investigating dental trauma, dental implants, biomaterials, maxillofacial surgery, and numerous other oral health issues [10, 11].

In recent years, research on impacted third molars has focused on improving extraction techniques [4], evaluating the effects of various drugs post-extraction [12, 13], assessing surgical difficulty [14], and examining the relationship between impacted third molars and mandibular fractures [15]. Mamoun et al. explored the use of dental elevators in clinical tooth extraction, discussing their types, applications, operational skills, and strategies for loosening and extracting teeth [16]. Additionally, Yan et al. used the finite element method to investigate how multiple variables (such as remaining tooth tissue thickness, slice gap width, depth of the dental elevator in the gap, position, width, and type of force) affect the efficiency of tooth slicing and trauma to surrounding tissues [17]. Marcus et al. proposed a method for extracting third molars using CBCT scan data combined with augmented reality technology, providing real-time three-dimensional images that help doctors better understand critical information during the extraction process [18]. Despite these advancements, current biomechanical analyses of root fractures remain limited.

This study aims to conduct biomechanical analysis to simulate the mechanical responses during the extraction of horizontal, mesioangular, and vertical impacted third molars in the mandible using dental elevators, and to analyze the stress distribution and fracture risk of the lever force and wedge force at three abduction angles (30°, 45°, 60°) and rotational moment along the X, Y, and Z axes. The fracture thresholds of three types of impacted third molar roots under different working conditions were obtained.

## Methods

### Materials

CBCT images: All CBCT images were obtained from the Stomatological Hospital of Guiyang City, underwent desensitization processing, and were saved as digital imaging and communications in medicine (DICOM) sequence files. The dataset included one case each of vertical, mesioangular and horizontal impacted third molars.

Extracted third molars: Six impacted third molars from the maxillofacial surgery Clinic of Guiyang Stomatological Hospital for preventive extraction have received medical ethical approval (GYSKLL-KY-20250117-01), regardless of age or sex. Inclusion criteria included complete impaction, completed apical development, no obvious surface cracks, and absence of various infectious diseases. The samples were stored in a 0.9% normal saline solution.

### Mechanical tensile testing of extracted impacted third molars

**Fig. 1:**
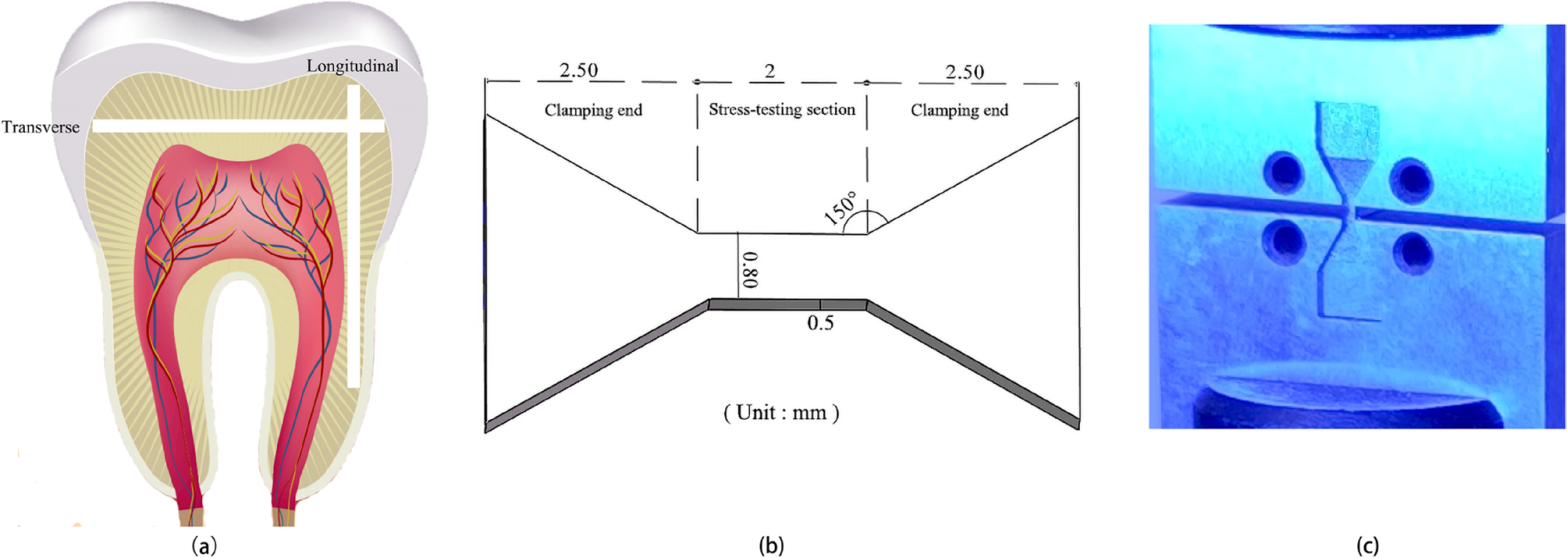
Processing drawing of the tooth specimen. Note: Figures **a**, **b**, and **c** respectively illustrate the transverse or longitudinal cutting path of the specimen, the dimensional requirements during machining, and the hanging-type clamps used in the tensile test

The arrangement of dentinal tubules determines the anisotropic structure of dentin in both longitudinal and transverse directions. Therefore, the cutting path was marked on the transverse or longitudinal direction of the extracted teeth (Fig. 1.a), and then fixed on the diamond-cutting machine (JGY6060). After cutting 0.5 mm thick slices, the specimens were processed into an overall “I” shape, with the clamping end being an inverted trapezoid and the stress-testing section between the two inverted trapezoids (Fig. 1.b). During the cutting process, water cooling was employed to minimize the accumulation of frictional heat. The specimens were considered qualified if the cutting surfaces were flat and free of cracks. A total of three transverse and three longitudinal specimens were obtained.

According to the Chinese national standard “Metallic Materials - Tensile Testing - Part 1: Method of Test at Room Temperature” (GB/T 228.1–2021), the tooth specimens were securely fastened to the mechanical testing machine (AGS-X 10KN) using hanging-type clamps (Fig. 1.c), ensuring that the loading direction was aligned with the main axis of the tooth slice. After preloading, the load was gradually applied at a tensile rate of 0.5 mm/min until the specimens fractured. The tensile data were recorded to generate the stress-strain curve and the displacement-load curve. Mechanical properties parameters such as elastic modulus, maximum load at fracture, ultimate strength, shear modulus, and Poisson’s ratio were determined. The average values of these parameters were used to evaluate the mechanical characteristics of the extracted impacted third molars.

### Finite element analysis

**Fig. 2:**
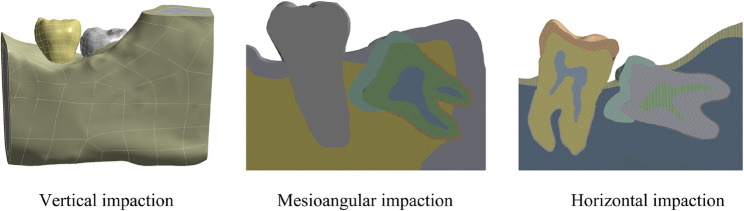
X_T format models of three types of impacted mandibular third molars

The CBCT datasets (3DeXam i) representing horizontal, vertical, and mesioangular impactions were imported into Mimics software. Based on the density and intensity characteristics of different tissues, the cortical bone, cancellous bone, second molar, and impacted third molar (including enamel, dentin, and pulp) were segmented layer by layer on three orthogonal planes. The 3D models were preliminarily reconstructed and optimized using the Calculate 3D, Smooth, and Wrap functions. These models were then exported in STL format and imported into Geomagic software to repair any holes and defects. Complete surface models were generated by drawing contour lines and converted into STP format files. The models were subsequently transformed into solid models in SolidWorks, where the periodontal ligament was constructed, and the model assembly was completed. Finally, the file was exported in X_T format (Fig. 2).

**Fig. 3:**
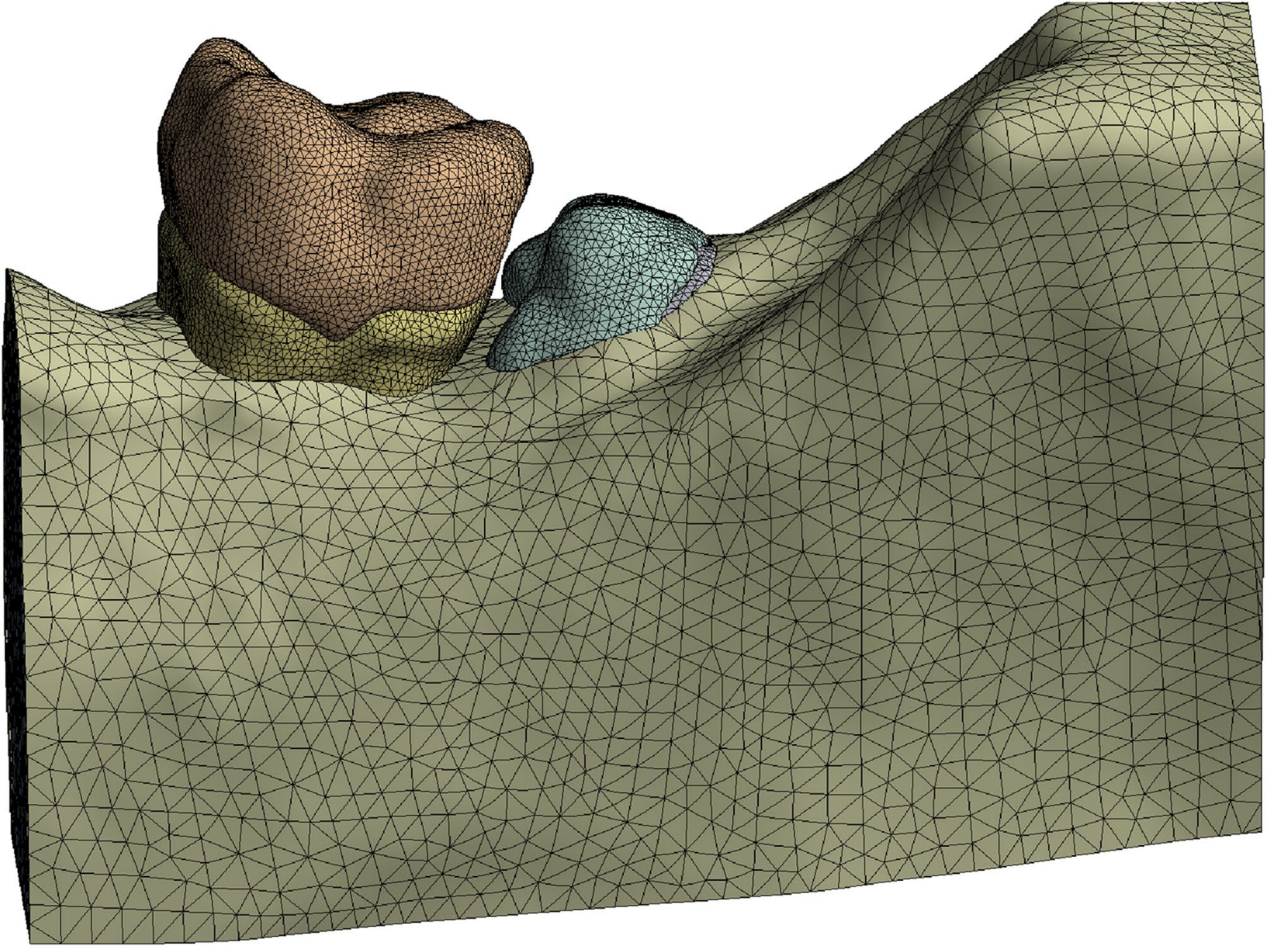
Grid model diagram

To ensure accurate material assignment, the X_T models were imported into Ansys Workbench for static structural analysis. Except for dentin, all other structures were modeled as continuous, linearly elastic, and isotropic materials. Material properties from the historical literature were manually input into the engineering database to complete the material assignment for each structure (Table 1). A three-dimensional 10-node tetrahedral element algorithm was used for meshing the model. Under the premise of ensuring mesh independence, conforming to geometric features, and meeting analysis accuracy, key areas of the impacted third molar were precisely processed (Table 1) (Fig. 3).

**Table 1:** Material properties and meshing of each tissue

Tissue	Elastic modulus (MPa)	Poisson’s ratio	Mesh size(mm)
Enamel [19]	84,100	0.33	0.3
Dental pulp [20]	2.07	0.45	0.3
Periodontal ligament [21]	68.9	0.45	0.1
Cortical bone [22]	13,700	0.3	1
Cancellous bone [22]	1370	0.3	1
dentin			0.3

The elastic modulus and Poisson’s ratio for dentin are not specified in this table. Relevant data can be found in Table 2, which presents the results of a previous tensile testing conducted on extracted third molars as part of this study

**Fig. 4:**
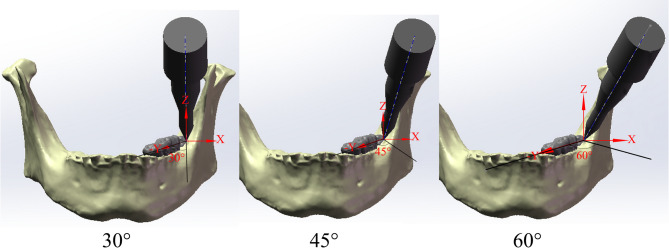
Three-dimensional diagram of the angle of dental elevators

The contact between tooth structures and cortical bone, cancellous bone, and periodontal membrane was set as frictional (µ = 0.2) [23, 24], while other contacts were defined as bonded contacts. Boundary conditions constrained mandibular displacement while permitting natural tooth movement. A local spatial coordinate system was defined, with the origin set at the buccal enamel-dentin junction. Specifically, the X-axis was oriented along the lingual-buccal direction, the Y-axis along the mesial-distal direction, and the Z-axis perpendicular to the jawbone. To emulate the procedures employed by oral surgeons during the use of dental elevators, the abduction angles for both the wedge force and lever force were configured at 30°, 45°, and 60°, while rotational moments were applied about the X, Y, and Z axes, respectively (Fig. 4).

In this coordinate system, the component values of the force (moment) on each axis represent both the magnitude and direction. The resultant force is calculated using Eq. (1):

1 $$\:\begin{array}{c}\text{F}\text{=}\sqrt{{\text{F}}_\text{X}^\text{2}\text{+}{\text{F}}_\text{Y}^\text{2}\text{+}{\text{F}}_\text{Z}^\text{2}}\end{array}$$

In this study, teeth are considered brittle materials. Therefore, the first strength criterion (maximum principal stress criterion) is adopted to numerically determine whether the material undergoes brittle fracture. The judgment condition is given by Eq. (2):

2 $$\sigma_1\leq\lbrack\sigma_{\mathrm b}\rbrack$$

Among them, $\:\sigma_1$ represents the maximum principal stress value obtained from the component test, and $\sigma_{\mathrm b}$ is the ultimate tensile strength of each structure [25].

The mechanical tensile test results are used to determine the anisotropy and ultimate tensile strength values of dentin.

## Results

### Test results of mechanical properties of extracted impacted third molars

**Fig. 5:**
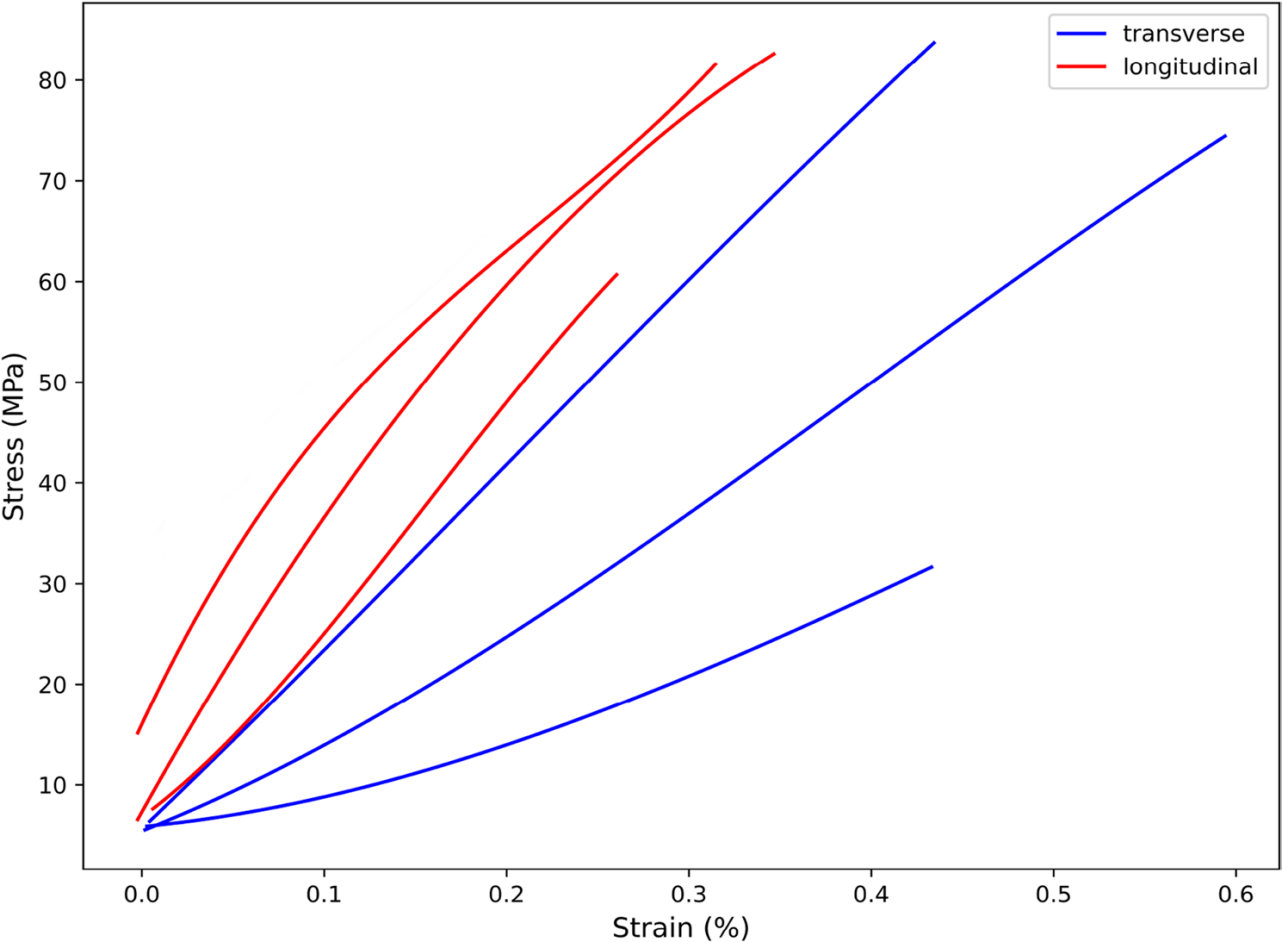
Transverse and longitudinal stress-strain curves

**Fig. 6:**
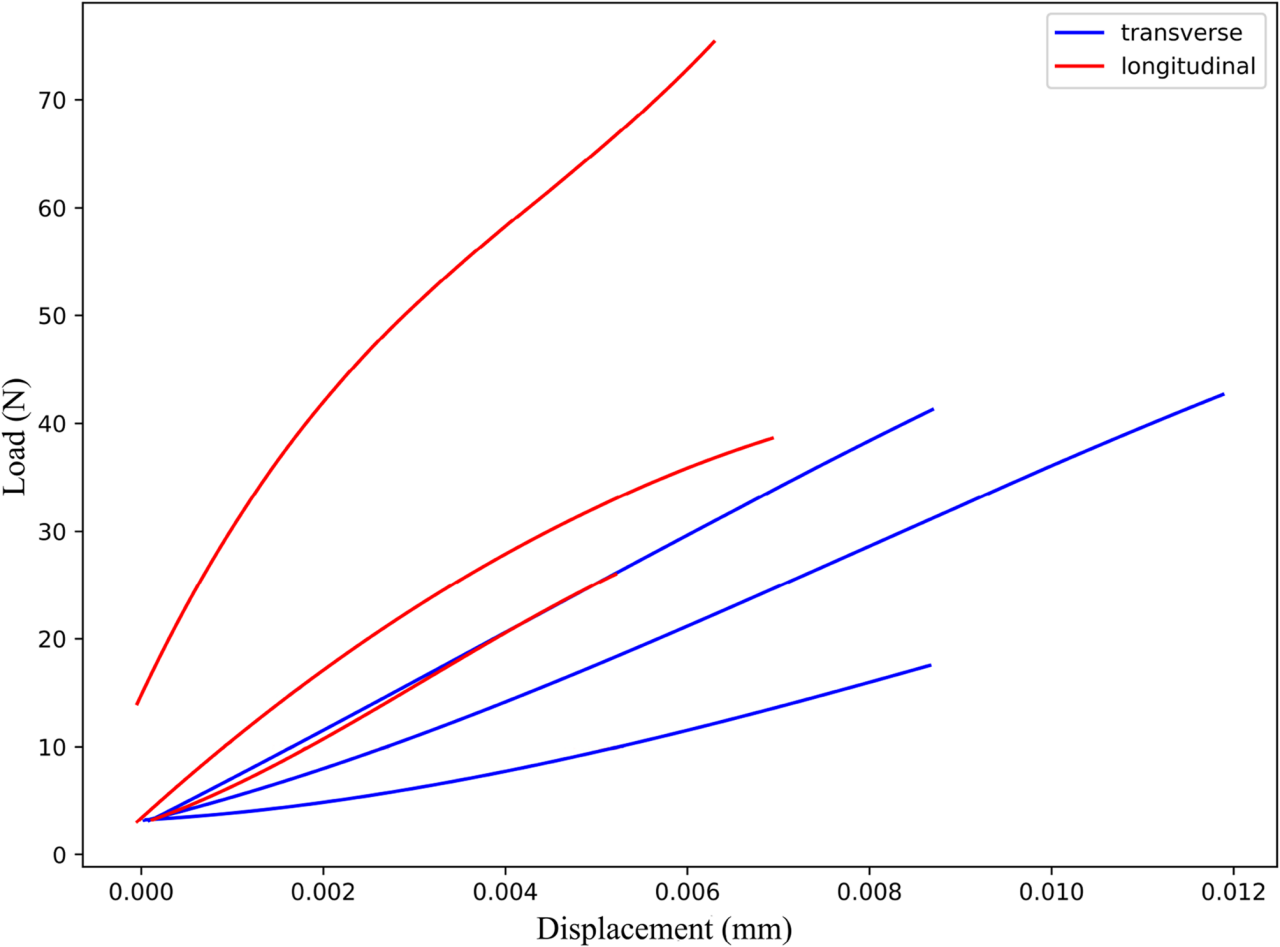
Displacement-load diagram in the transverse and longitudinal directions

The data obtained from the tests were plotted and smoothed using polynomial fitting, as shown in Figs. 5 and 6.

As shown in Fig. 5, under transverse loading, the curve exhibits a relatively small slope, with the maximum stress values varying across different specimens. The average elastic modulus is 14.32 GPa, and the ultimate tensile strength is 63.28 MPa. Under longitudinal loading, dentin displays a higher stress response, fracturing at strain between 0.25% and 0.35%, and shows strong elastic behavior. The average elastic modulus is 21.53 GPa, and the ultimate tensile strength is 74.54 MPa. Compared to transverse loading, longitudinal loading demonstrates higher tensile strength and greater deformation capacity, whereas transverse loading tends to be more brittle. However, both loading conditions result in brittle fracture.

From Fig. 6, it can be observed that under transverse loading, the curve shows a relatively linear growth trend, with varying maximum load values among different samples, averaging 30.27 N. Under longitudinal loading, the average load at which the tooth slices fracture is 46.3 N, significantly higher than the fracture load under transverse loading. The material can withstand a higher load without significant displacement, and the curve does not show a clear linear relationship, indicating better mechanical stability and stronger load-bearing capacity.

Additionally, the anisotropic parameters of dentin were calculated based on the test data, as presented in Table 2.

**Table 2:** Anisotropic parameters of dentin

Parameter	E_X_ (GPa)	E_Y_ (GPa)	E_Z_ (GPa)	ν_XY_	ν_YZ_	ν_XZ_	G_XY_ (GPa)	G_YZ_ (GPa)	G_XZ_ (GPa)	UTS (MPa)
Value	14.32	14.32	21.53	0.3	0.33	0.3	5.51	8.09	5.51	63.28

### Fracture threshold 

**Fig. 7:**
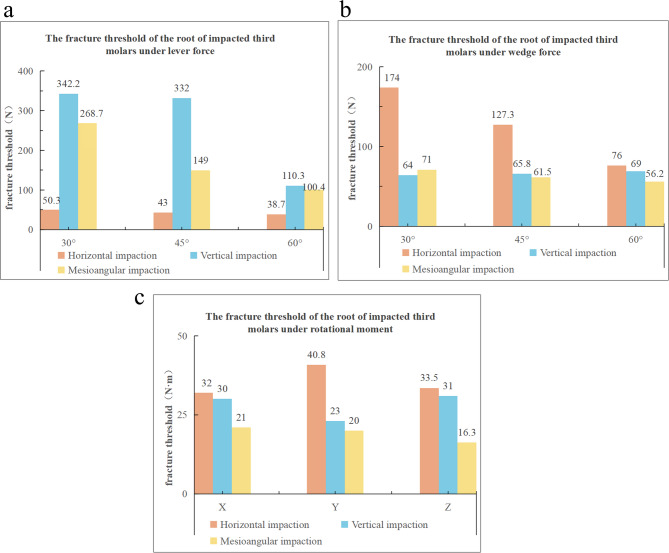
Bar chart of fracture threshold results

As shown in Fig. 7, the fracture thresholds of roots in different types of impacted third molars vary under different working conditions. Under lever force, the fracture threshold of horizontal impaction is minimally affected by changes in the force application angle, whereas those of vertical and mesioangular impactions exhibit a negative correlation with the force application angle (Fig. 7a). Under wedge force, the fracture threshold of horizontal impaction shows a negative correlation with the force application angle, while that of vertical impaction demonstrates a slight positive correlation, though not significant. In contrast, the fracture threshold of mesioangular impaction is negatively correlated with the force application angle (Fig. 7b). Under rotational moment, the fracture thresholds of all three impaction types remain relatively stable across different directions (Fig. 7c). Overall, under lever force, vertical impaction is the least prone to fracture, while horizontal impaction is the most susceptible. Under wedge force, horizontal impaction is the least likely to fracture. Under rotational moment, horizontal impaction is less likely to fracture in all three directions, mesioangular impaction is more prone to fracture along the Z-axis, and vertical impaction is most susceptible to fracture along the Y-axis.

### Stress results of impacted third molars and surrounding tissues

**Fig. 8:**
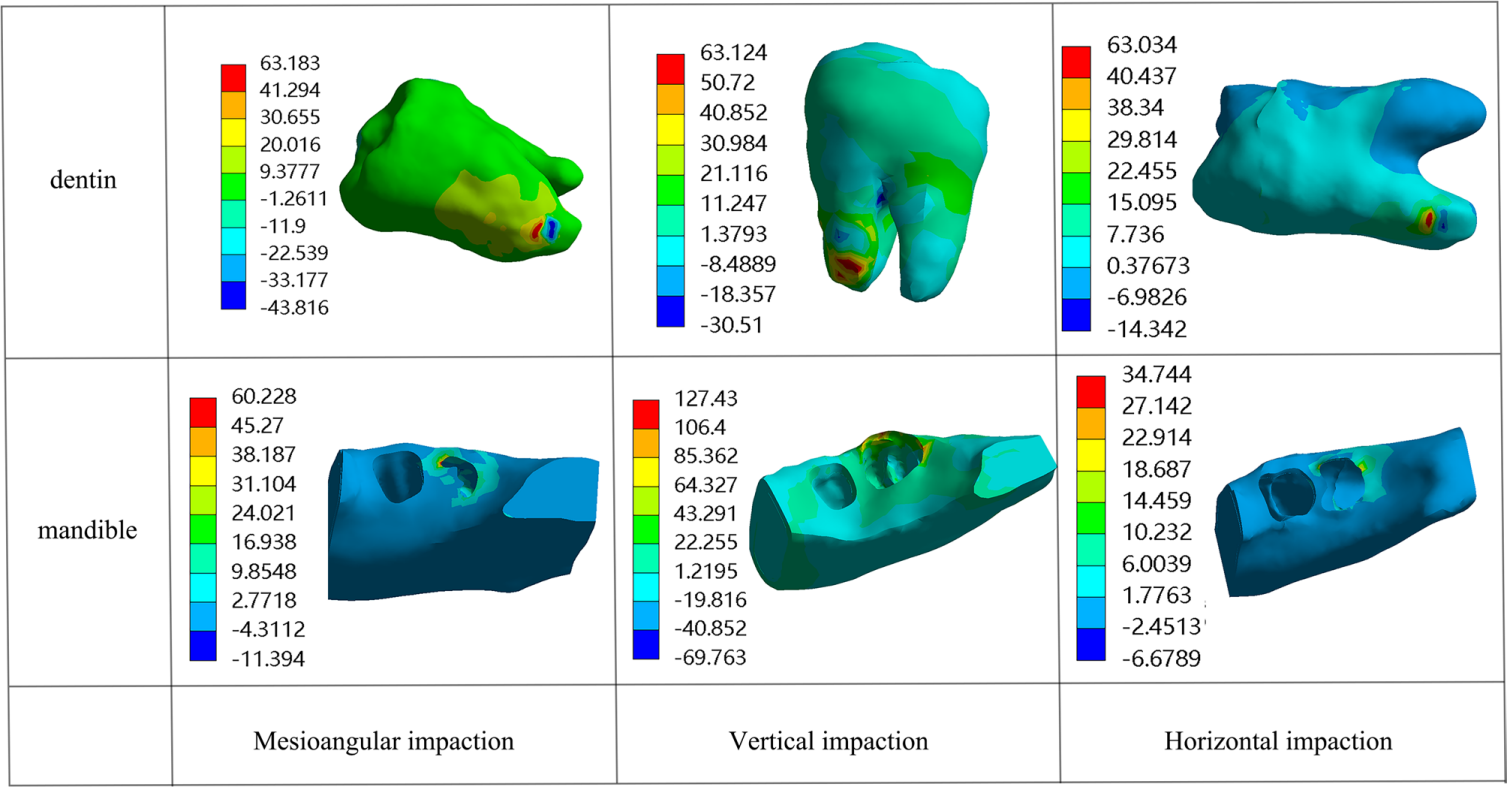
Stress distribution of three types of impactions under a 45° lever force

As illustrated in Fig. 8 and additional file 1, under the application of lever force, as the force angle increases, the stress changes in the pulp of horizontally impacted teeth are not significant. However, the stress in the enamel, periodontal ligament, adjacent teeth, and mandible decreases, while the compressive stress in the dentin undergoes significant changes and decreases. For mesioangular impaction, the stress on all tissues decreased, with a more pronounced reduction in dentin compressive stress. In the case of vertical impaction, the stress in the periodontal ligament decreases, while the stress changes in other tissues are significant but irregular. Stress concentration is particularly evident in the mesial root and alveolar ridge regions.

When wedge force is applied, as the force angle increases, for horizontal impaction, the compressive stress in the dental pulp and dentin significantly decreases, while the stress in other tissues also shows a decreasing trend, though less pronounced. For mesioangular impaction, the compressive stress in the enamel, dentin, and adjacent teeth changes significantly and decreases, whereas the stress in the dental pulp increases. The stress in the periodontal ligament changes irregularly, and the tensile and compressive stress in the mandible exhibit opposite trends. In vertical impaction, the stress in the dental pulp, dentin, and periodontal ligament does not change significantly, but the compressive stress in the enamel and mandible decreases significantly, and the stress in the adjacent teeth also decreases. Stress concentration areas are similarly observed in the mesial root and alveolar ridge regions.

Under the application of rotational moment, for all three types of impacted third molars, the compressive and tensile stress in each tissue shows no obvious relationship with the rotation direction. Nevertheless, it is noteworthy that stress concentrations are predominantly identified in the mesial root regions.

## Discussion

This study is the first to quantitatively analyze the effects of lever force, wedge force, and rotational moment exerted by dental elevators at different abduction angles on the fracture threshold of roots in various types of impacted third molars, as well as to investigate the stress distribution in the surrounding tissues and identify fracture risk areas. The results demonstrate that as the dental elevator’s abduction angle increases, horizontal mandible impacted third molars demonstrate the lowest fracture resistance under lever forces (38.7–50.3 N), while vertical impactions exhibit the highest thresholds (110.3–342.2 N). For mesioangular mandible impacted third molars, the root fracture threshold is negatively correlated with the angle of the applied lever force. Under wedge forces, horizontal impactions show an inverse correlation between fracture thresholds and abduction angles (76–174 N), while vertical impactions display a gradual but insignificant increase in fracture thresholds. The rotational moment remains relatively stable across all three directions for horizontal, vertical, and mesioangular impacted third molars. Additionally, periradicular tissue stress decreases with increasing abduction angles under lever and wedge forces (*p* < 0.05), but no significant directional correlation is observed under rotational moments.

Currently, studies on the mechanical behavior of dental elevators during tooth extraction are relatively limited, with most research focusing on forceps extraction or the clinical application of dental elevators [16, 26], lacking systematic mechanical analysis and comprehensive data comparison. However, our findings indicate that stress concentration predominantly occurs in the mesial root region, which aligns with clinical observations. The mesial root, owing to its anatomical features (such as curvature and bifurcation) and proximity to adjacent teeth, is more susceptible to becoming a high-stress concentration zone during tooth extraction. This unique morphological characteristic results in uneven stress distribution and elevates the risk of root fracture, particularly at mechanically vulnerable sites like curved or bifurcated regions. Dental elevators separate the tooth root from the alveolar bone through wedge, lever, and rotational actions. By combining the fracture thresholds of the root under different working conditions, the force applied by the dentist’s hand can be deduced. For example, when the dental elevator is positioned at an angle θ and the fracture threshold is F1, then the wedge force (the component force along the direction of the wedge entry) applied by the dentist should not exceed (F1/sinθ) N. Similarly, when a rotational moment is applied to an impacted third molars, and the fracture threshold is M, and the distance from the hypothenar of the palm to the rotation center is L1 mm, then the force used by the dentist should not exceed (M/L1) N. Although this study used ideal isotropic parameters for tissues other than dentin, which may lead to less accurate stress distribution in these tissues, the primary focus on the fracture threshold of the third molars root and stress concentration phenomenon means that this simplification has a minimal impact on the overall conclusion. Future research can follow the actual characteristics of oral anatomy, such as the nonlinear mechanical behavior of the periodontal ligament [27], and the anisotropic properties of the alveolar bone [28], to achieve more precise mechanical simulation.

Prophylactic extraction of impacted third molars is a common yet challenging procedure in oral surgery. In clinical practice, quantifying the fracture threshold of tooth roots helps oral surgeons to rationally design the force application type and magnitude during extracting, taking into account the type of impaction (such as horizontal, vertical, and mesial impaction) and mechanical characteristics. This ensures that the operation is within the safe range of the tooth root fracture threshold, thereby minimizing the risk of tooth root fracture complications and enhancing surgical success rates. Furthermore, these research findings provide an important reference for subsequent development of surgical path planning and intelligent reasoning systems by the research group, and establish a theoretical foundation for the advancement of intelligent oral medical equipment (such as intelligent tooth forceps and tooth extraction robots). For instance, by integrating fracture threshold data and stress distribution patterns with three-dimensional path planning algorithms, the system can automatically identify and avoid critical anatomical structures (such as tooth roots, alveolar bone, and adjacent teeth), enabling the formulation of optimal extraction paths and force application strategies to minimize intraoperative complications and improve surgical success rates. In addition, conventional dental elevators may be enhanced with intelligent features by integrating pressure sensors and upper-limit force warning systems. When the applied force exceeds the predefined safety threshold, the device can issue real-time alerts to help clinicians perceive and control the force more precisely.

This study has certain limitations. The tensile test did not account for the gender, age, or race of the extracted teeth, which may result in insufficient diversity and representativeness of the sample sources. The singular mechanical test approach may limit the comprehensive assessment of the mechanical properties of impacted third molars and the universality of the findings. Additionally, in the finite element analysis, only one representative case was selected for each type of impaction, which could not account for inter-individual anatomical variability and restrict the broader clinical applicability of the findings. Future research could conduct comparative analyses across different racial groups and a wider range of impaction cases to further elucidate the biomechanical response characteristics of impacted third molars under various loading conditions. By establishing a comprehensive database of the mechanical characteristics of impacted teeth covering a broader population, a more precise theoretical basis can be provided for clinical operations and the optimization of tooth extraction instruments and techniques can be promoted.

## Conclusions

Based on the anisotropic characteristics of dentin, this study explored the influence of different abduction angles of dental elevators on the root fracture threshold and stress distribution in the surrounding tissue of impacted mandibular third molars. The findings provide an important theoretical basis for oral surgeons in extracting impacted third molars and for the subsequent development of surgical path planning and intelligent reasoning systems by the research group:

- For vertical impaction (safety margin > 100 N), the clinical plan should prioritize the use of lever force for extraction; for horizontal impaction, wedge and rotational forces are more effective.
- Stress concentration mainly occurs at the mesial root of the tooth.
- As the abduction angle increases, the fracture threshold and tissue stress under lever force decrease. Under wedge force, except for a slight increase in the threshold for vertical impaction, the rest is consistent with that under lever force; under rotational moment, the fracture threshold remains stable, tissue stress shows no directional dependence.

## Supplementary Information

Additional file 1. In the "Additional Files 1" folder, there are 9 image files in PNG format. The naming rules for each file are as follows: the section before the underscore indicates the type of force applied by the dental elevator used, while the section after the underscore indicates the corresponding mandible impacted third molar type.

## Data Availability

Data is provided within the manuscript or supplementary information files.

## Abbreviations

CBCT: Cone beam computed tomography
FEA: Finite element analysis
DICOM: Digital imaging and communications in medicine
