# Supplementary figures and images for: Fracture threshold of tooth roots and stress analysis of surrounding tissue during the extraction of impacted mandibular third molars using dental elevators
Source: BMC Oral Health. 2025 Oct 8;25:1573. doi: 10.1186/s12903-025-06744-2 (PMC12509397; doi:10.1186/s12903-025-06744-2)

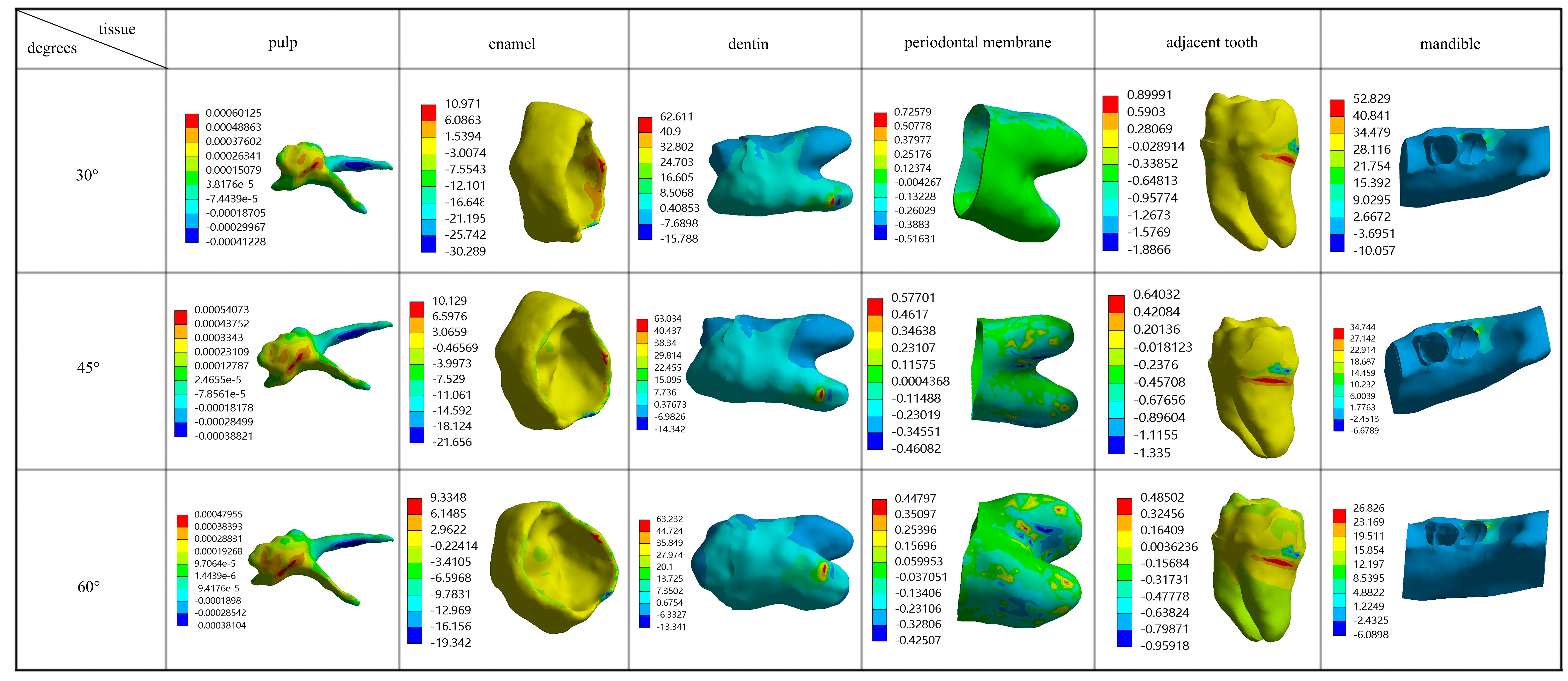

Supplement: Supplementary file 1 — Additional file 1. In the "Additional Files 1" folder, there are 9 image files in PNG format. The naming rules for each file are as follows: the section before the underscore indicates the type of force applied by the dental elevator used, while the section after the underscore indicates the corresponding mandible impacted third molar type. [file 12903_2025_6744_MOESM1_ESM.zip › Additional file 1/lever force_horizontal impaction.png]

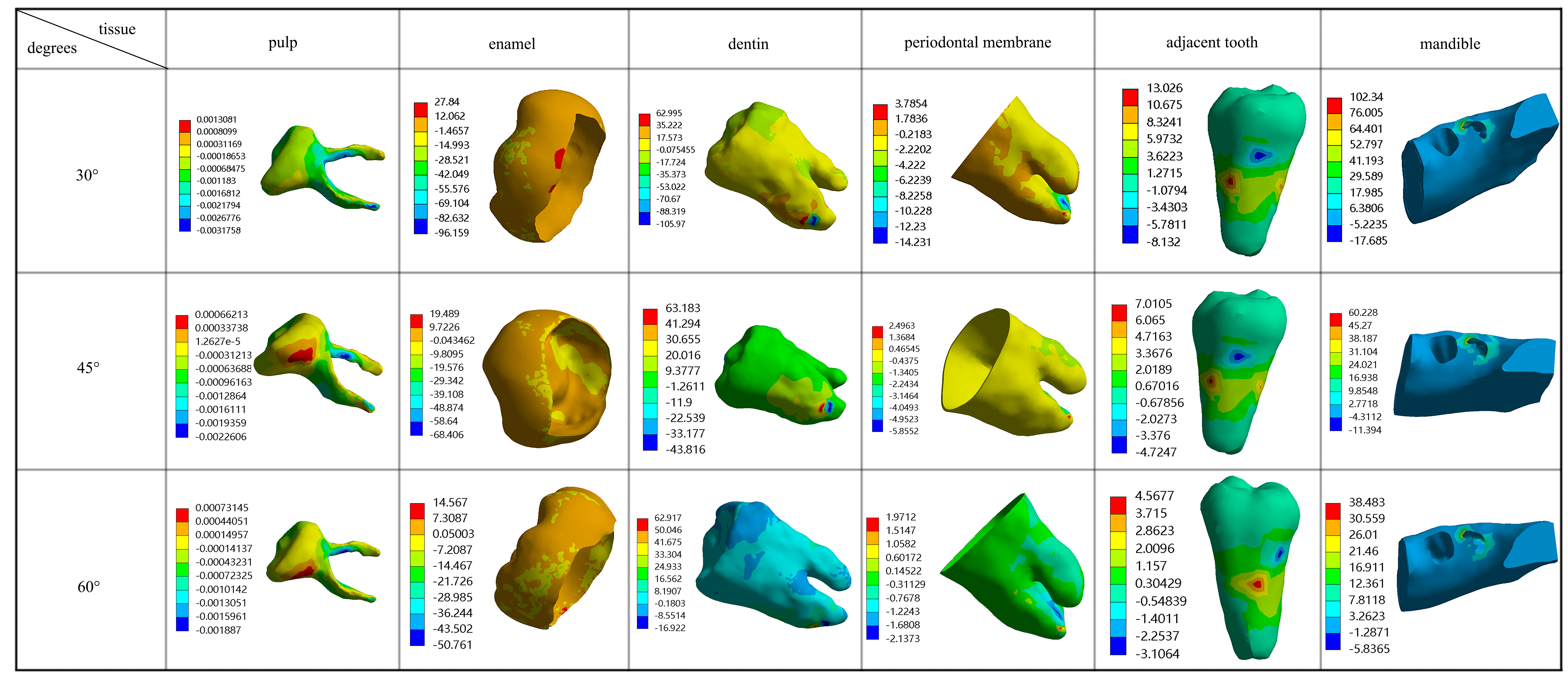

Supplement: Supplementary file 1 — Additional file 1. In the "Additional Files 1" folder, there are 9 image files in PNG format. The naming rules for each file are as follows: the section before the underscore indicates the type of force applied by the dental elevator used, while the section after the underscore indicates the corresponding mandible impacted third molar type. [file 12903_2025_6744_MOESM1_ESM.zip › Additional file 1/lever force_mesioangular impaction.png]

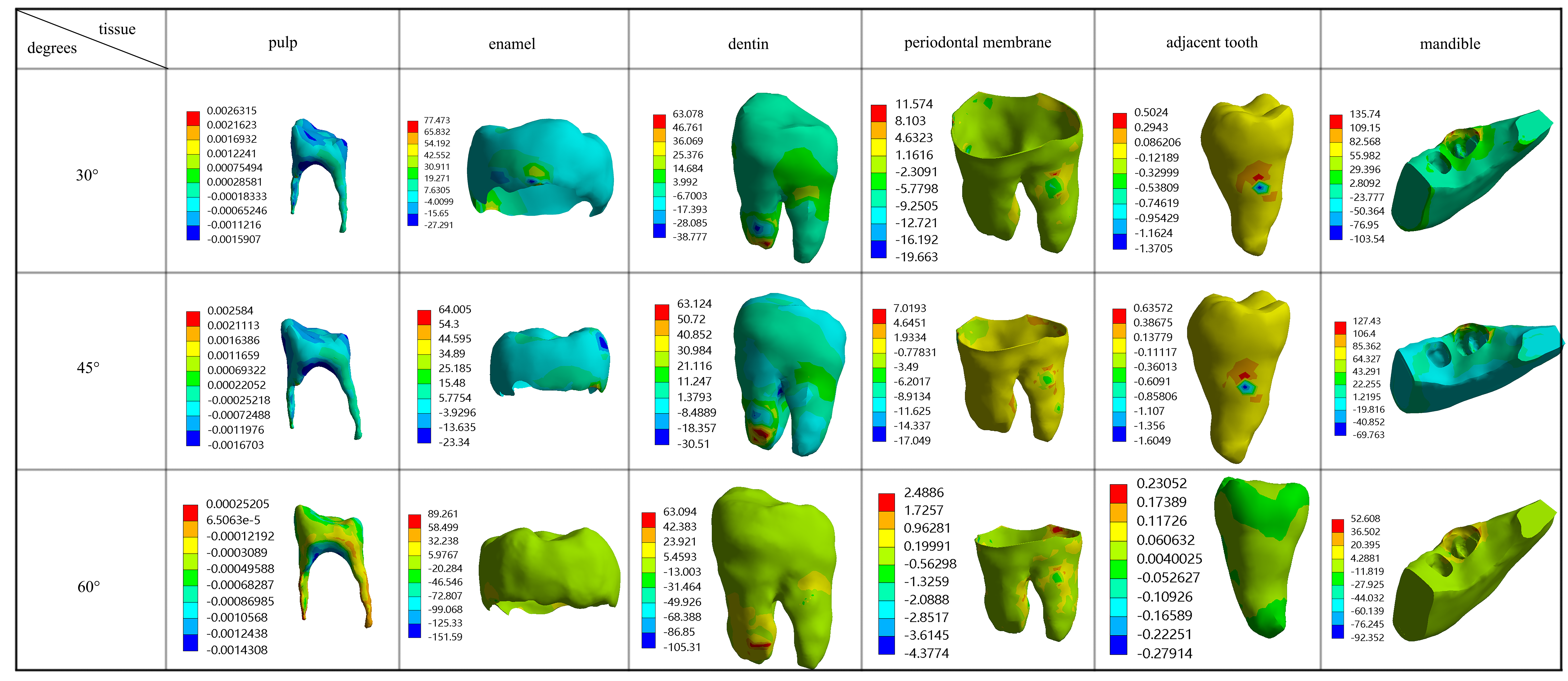

Supplement: Supplementary file 1 — Additional file 1. In the "Additional Files 1" folder, there are 9 image files in PNG format. The naming rules for each file are as follows: the section before the underscore indicates the type of force applied by the dental elevator used, while the section after the underscore indicates the corresponding mandible impacted third molar type. [file 12903_2025_6744_MOESM1_ESM.zip › Additional file 1/lever force_vertical impaction.png]

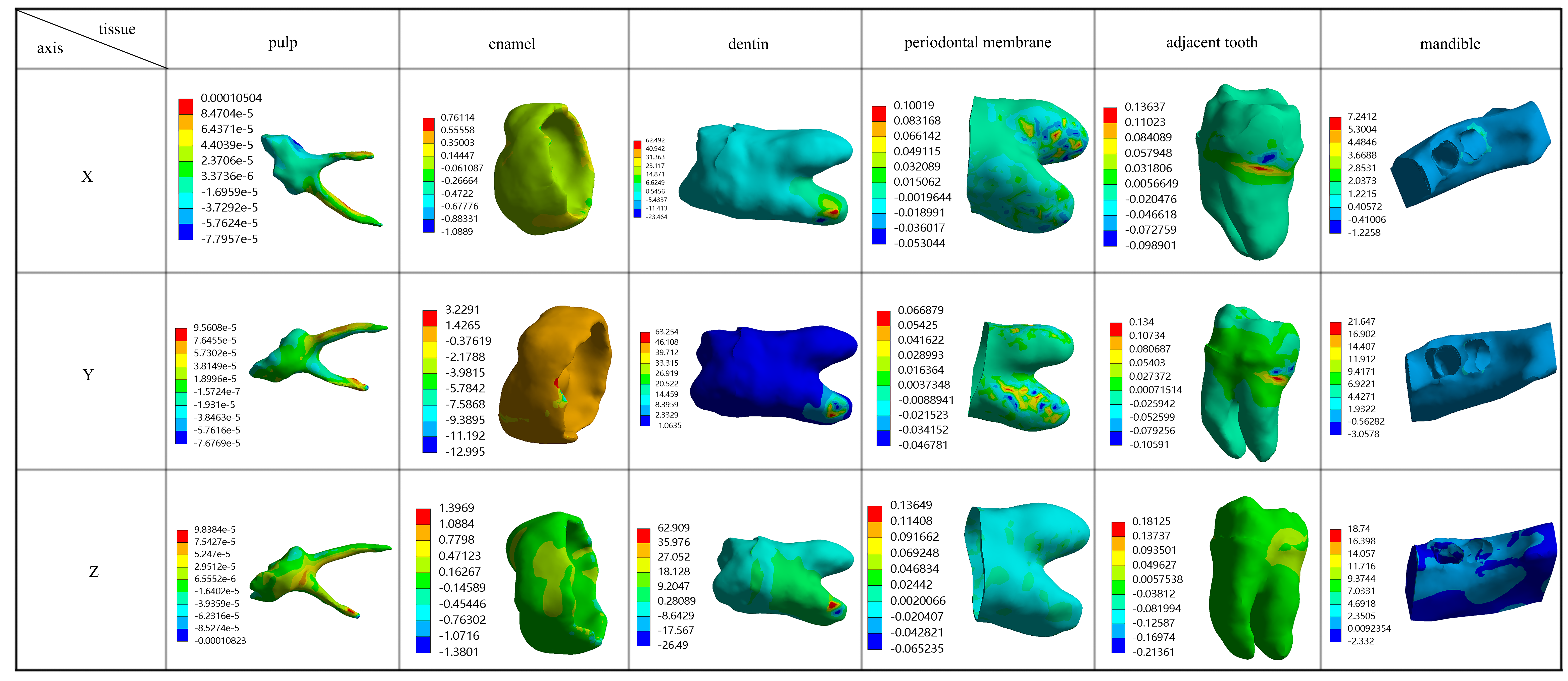

Supplement: Supplementary file 1 — Additional file 1. In the "Additional Files 1" folder, there are 9 image files in PNG format. The naming rules for each file are as follows: the section before the underscore indicates the type of force applied by the dental elevator used, while the section after the underscore indicates the corresponding mandible impacted third molar type. [file 12903_2025_6744_MOESM1_ESM.zip › Additional file 1/rotational moment_horizontal impaction.png]

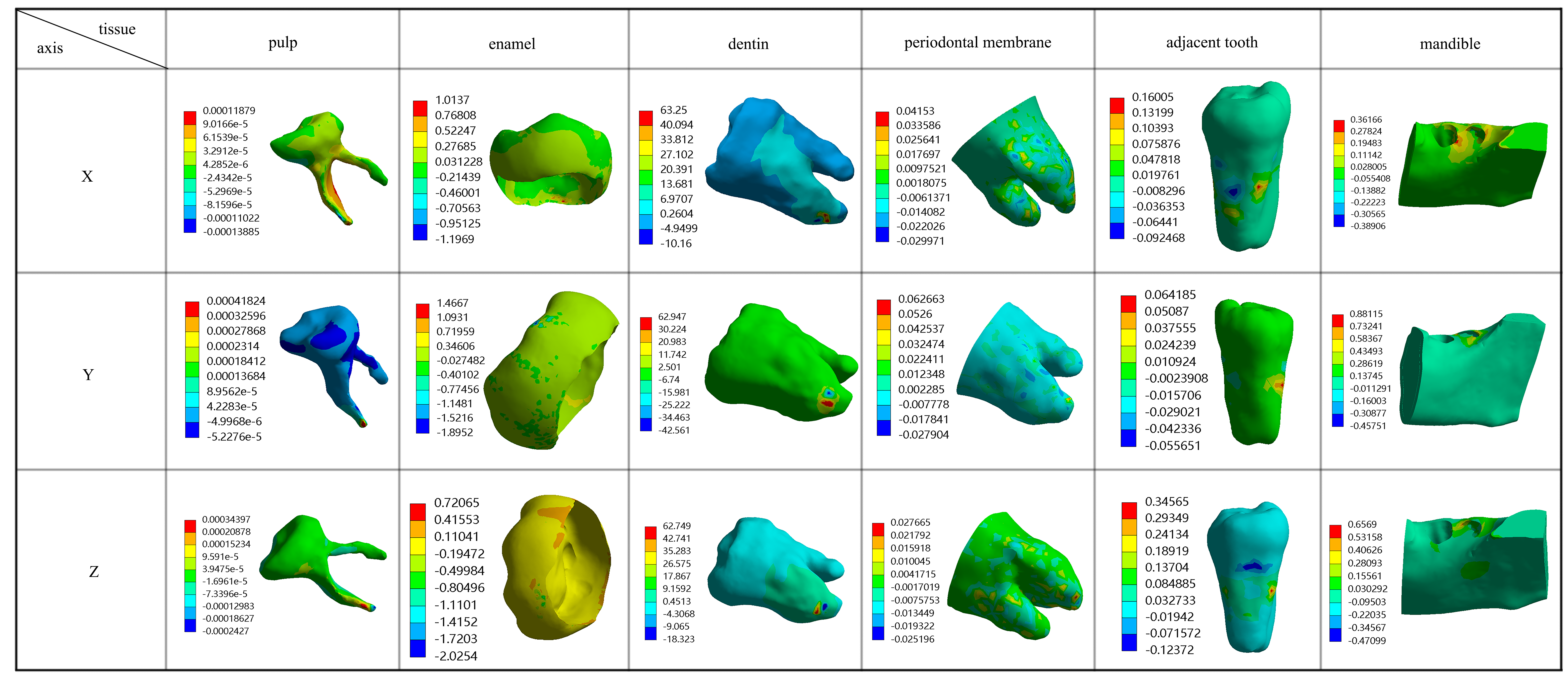

Supplement: Supplementary file 1 — Additional file 1. In the "Additional Files 1" folder, there are 9 image files in PNG format. The naming rules for each file are as follows: the section before the underscore indicates the type of force applied by the dental elevator used, while the section after the underscore indicates the corresponding mandible impacted third molar type. [file 12903_2025_6744_MOESM1_ESM.zip › Additional file 1/rotational moment_mesioangular impaction.png]

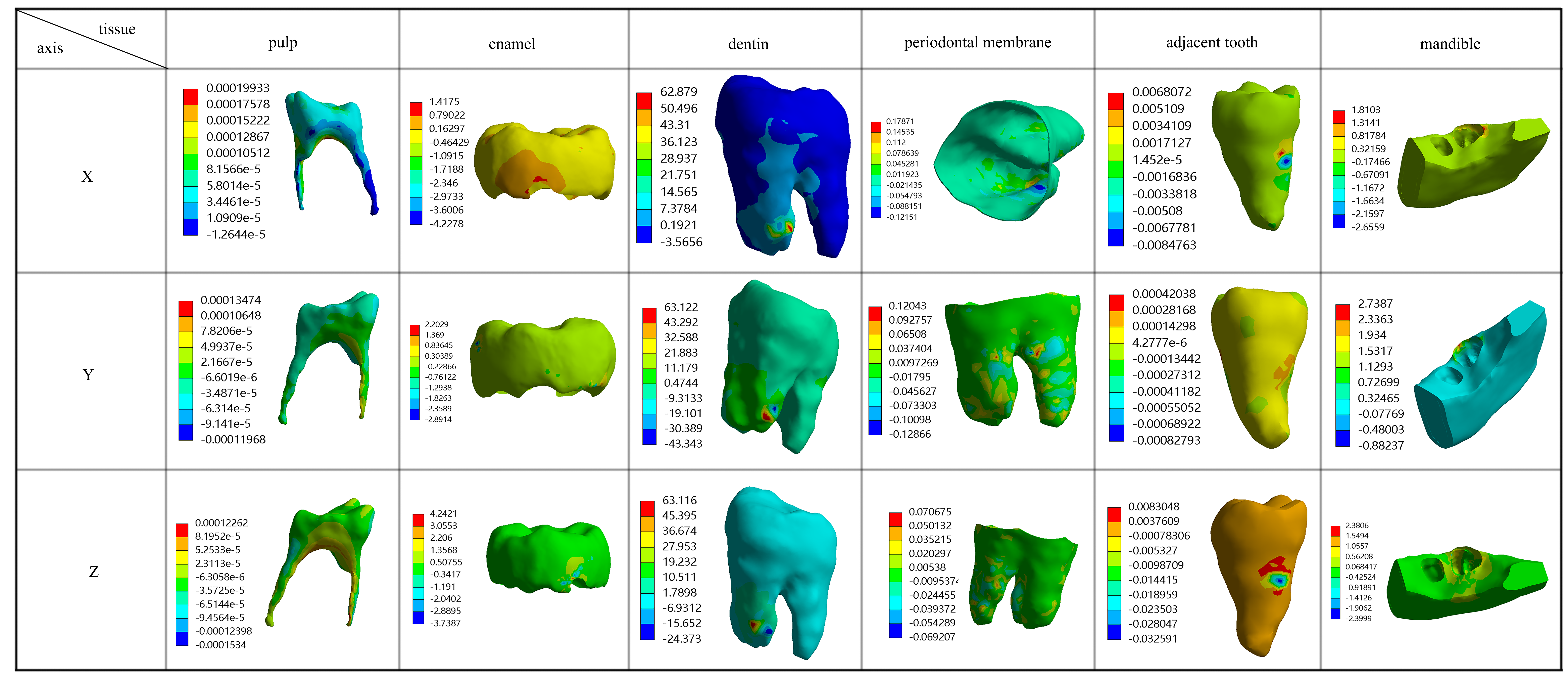

Supplement: Supplementary file 1 — Additional file 1. In the "Additional Files 1" folder, there are 9 image files in PNG format. The naming rules for each file are as follows: the section before the underscore indicates the type of force applied by the dental elevator used, while the section after the underscore indicates the corresponding mandible impacted third molar type. [file 12903_2025_6744_MOESM1_ESM.zip › Additional file 1/rotational moment_vertical impaction.png]

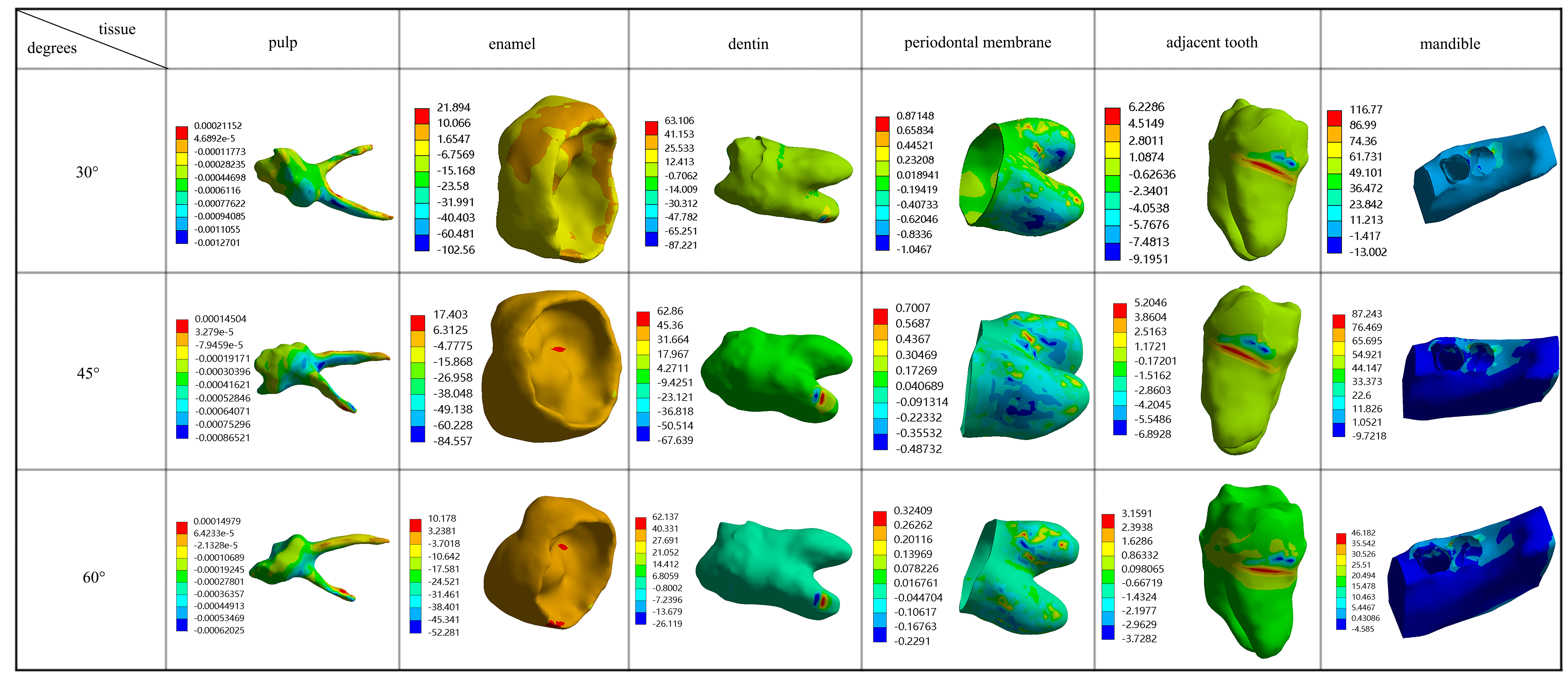

Supplement: Supplementary file 1 — Additional file 1. In the "Additional Files 1" folder, there are 9 image files in PNG format. The naming rules for each file are as follows: the section before the underscore indicates the type of force applied by the dental elevator used, while the section after the underscore indicates the corresponding mandible impacted third molar type. [file 12903_2025_6744_MOESM1_ESM.zip › Additional file 1/wedge force_horizontal impaction.png]

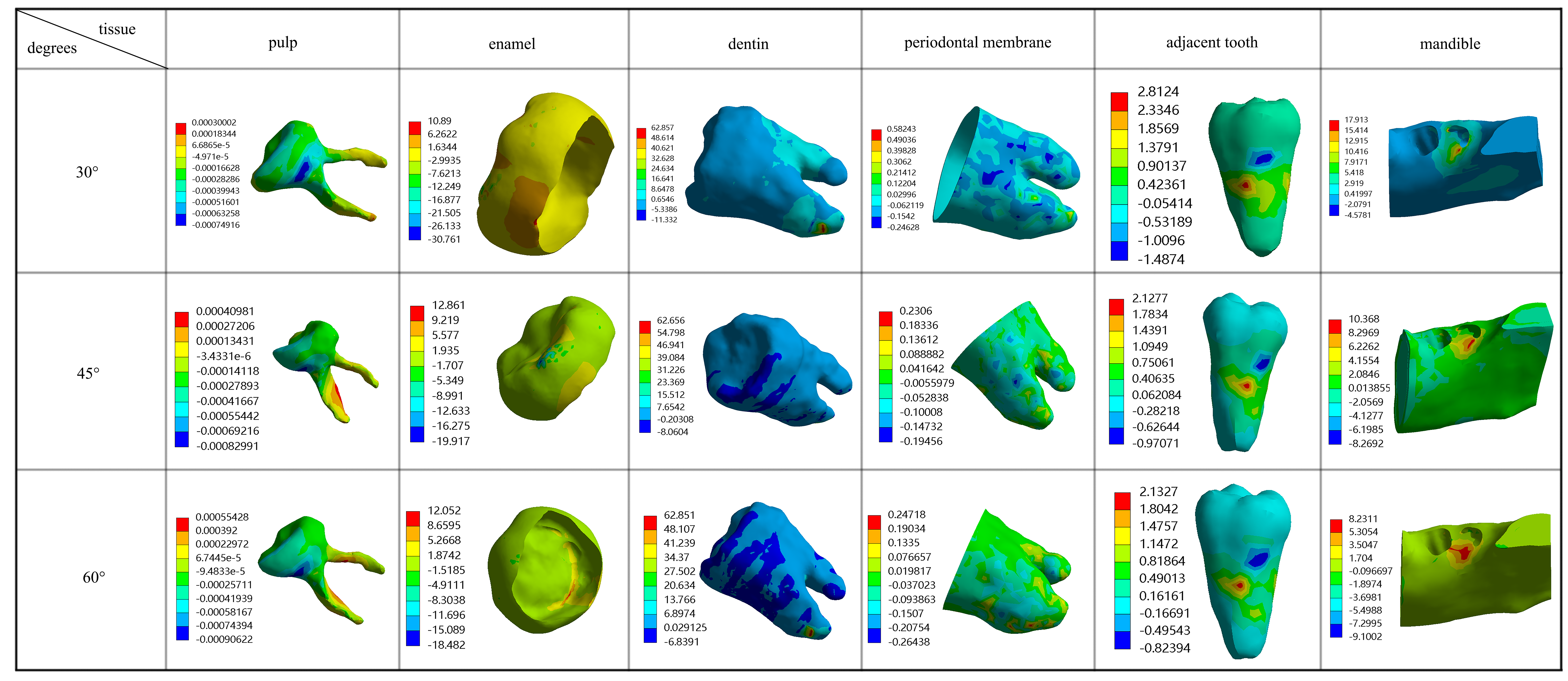

Supplement: Supplementary file 1 — Additional file 1. In the "Additional Files 1" folder, there are 9 image files in PNG format. The naming rules for each file are as follows: the section before the underscore indicates the type of force applied by the dental elevator used, while the section after the underscore indicates the corresponding mandible impacted third molar type. [file 12903_2025_6744_MOESM1_ESM.zip › Additional file 1/wedge force_mesioangular impaction.png]

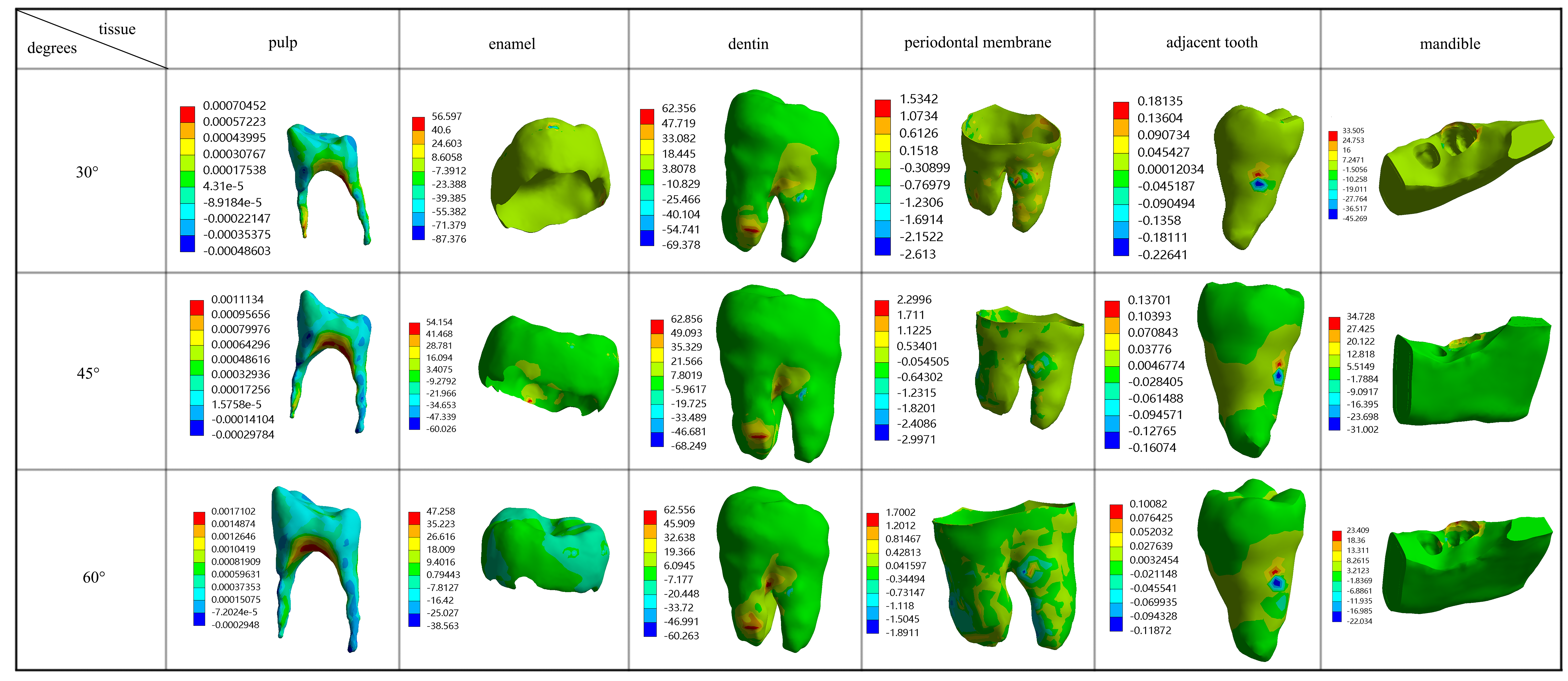

Supplement: Supplementary file 1 — Additional file 1. In the "Additional Files 1" folder, there are 9 image files in PNG format. The naming rules for each file are as follows: the section before the underscore indicates the type of force applied by the dental elevator used, while the section after the underscore indicates the corresponding mandible impacted third molar type. [file 12903_2025_6744_MOESM1_ESM.zip › Additional file 1/wedge force_vertical impaction.png]
